# Landscape Structure Shapes Habitat Finding Ability in a Butterfly

**DOI:** 10.1371/journal.pone.0041517

**Published:** 2012-08-01

**Authors:** Erik Öckinger, Hans Van Dyck

**Affiliations:** 1 Department of Ecology, Swedish University of Agricultural Sciences, Uppsala, Sweden; 2 Biodiversity Research Centre, Earth and Life Institute, Université catholique de Louvain (UCL), Louvain-la-Neuve, Belgium; University Copenhagen, Denmark

## Abstract

Land-use intensification and habitat fragmentation is predicted to impact on the search strategies animals use to find habitat. We compared the habitat finding ability between populations of the speckled wood butterfly (*Pararge aegeria* L.) from landscapes that differ in degree of habitat fragmentation. Naïve butterflies reared under standardized laboratory conditions but originating from either fragmented agricultural landscapes or more continuous forested landscapes were released in the field, at fixed distances from a target habitat patch, and their flight paths were recorded. Butterflies originating from fragmented agricultural landscapes were better able to find a woodlot habitat from a distance compared to conspecifics from continuous forested landscapes. To manipulate the access to olfactory information, a subset of individuals from both landscape types were included in an antennae removal experiment. This confirmed the longer perceptual range for butterflies from agricultural landscapes and indicated the significance of both visual and olfactory information for orientation towards habitat. Our results are consistent with selection for increased perceptual range in fragmented landscapes to reduce dispersal costs. An increased perceptual range will alter the functional connectivity and thereby the chances for population persistence for the same level of structural connectivity in a fragmented landscape.

## Introduction

Human-dominated landscapes are characterized by high levels of habitat loss and fragmentation [1]. In highly fragmented landscapes, dispersal between local populations is crucial for population persistence [2]–[3], but dispersal is also assumed to be costly in terms of mortality risks, energetic costs or both [4]. Dispersal is likely to be particularly costly in landscapes under intensive human land use, where distances between habitat patches are large [4]. Land-use intensification in the landscape matrix may impact on the search strategies animals use to find habitat [5]–[7]. Variation in perceptual range, i.e. the distance at which an animal can detect objects in the landscape [8] may strongly affect the trajectories of animals across anthropogenic landscapes, and thereby the costs of dispersal [6]–[7], [9]. The ability of species to adapt their search strategies following environmental changes is likely to significantly influence their survival in intensively modified landscapes. Simulation models suggest that animals moving through the landscape could reduce dispersal costs by adjusting habitat search strategies and abilities [10], but this has not been tested empirically.

Species with different life histories use different types on information to orientate while moving. Nocturnal insects, for example most moths (Lepidoptera), heavily depend on olfactory cues at longer distances to find habitat and mates [11], but diurnal Lepidoptera, including butterflies, are assumed to rely much more on visual cues with altered eye structure compared to moths [12]. However, butterflies also use olfactory cues to identify key aspects of habitat quality from a short distance [13]–[14]. It is, however, not known how important olfactory information is for butterflies at larger spatial scales, and has therefore largely been ignored in landscape ecological studies.

Although abundances and distributions of many plant and animal species have recently declined as a result of intensified human land use [15]–[16], some species maintain stable populations or even increase in numbers, possibly due to their high levels of life history plasticity [17]. The Speckled wood butterfly (*Pararge aegeria* L.) is one such example of species that are highly successful in landscapes under intense human use [17]. *P. aegeria* is primarily a woodland species, but in NW-Europe it also occurs in landscape types dominated by other land uses (i.e. agricultural landscapes with scattered small woodlots and hedgerows).

**Figure 1:**
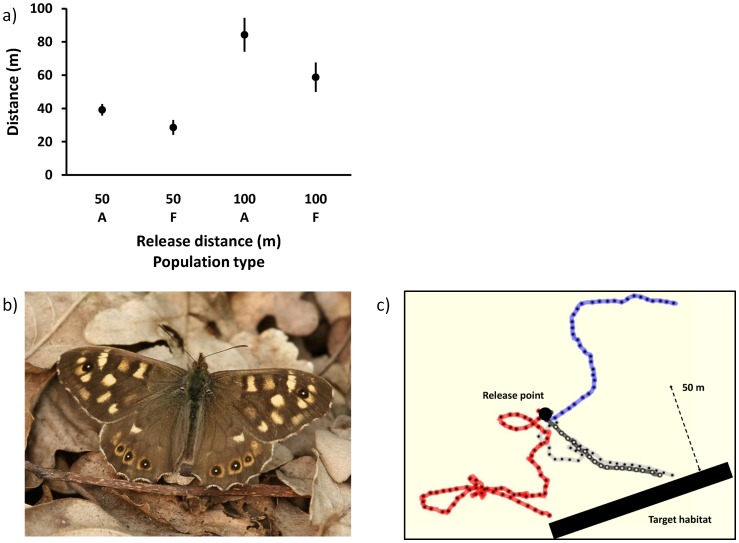
a) Butterflies from populations in forest landscapes (F) initiate a linear flight path directed to the target habitat at shorter distances than butterflies from populations in agricultural landscapes (A). This holds for butterflies released at both 50 and 100 m. Mean ± SE are shown. **b**) Male of the Speckled wood butterfly *Pararge aegeria* L. (Photo by M. Jacobs, Belgium), **c**) Examples of observed flight paths. Blue: A butterfly that did not reach the target habitat, Black: A butterfly that was flying towards the target habitat from take-off, Red and grey: Butterflies that were first undertaking different types of search behavior, and eventually flew towards the target habitat.

We compared habitat finding ability in *P. aegeria* butterflies that originated from populations in continuous forested landscapes with butterflies originating from populations in fragmented agricultural landscapes with some woodlots and hedgerows [18] to test the if landscape structure selects for altered perceptual range. A previous experiment with wild-caught butterflies indicated that there are indeed landscape-dependent differences in habitat-finding ability in this species, at least in males [19]. Here, we build on this previous experiment and use butterflies of both sexes and from replicated populations in each landscape type reared under common-garden lab conditions to test if the landscape of origin has heritable effects on the habitat-finding ability. Further, we tested the relative importance of visual and olfactory cues on the habitat finding ability of butterflies by experimentally removing the antennae from butterflies from both landscape types. Based on our results we conclude that 1) individuals originating from fragmented landscapes have a better habitat-finding ability than individuals from more continuous landscapes, consistent with a selection to reduce dispersal costs, and 2) butterflies use a combination of visual and olfactory information to locate suitable habitat patches.

**Figure 2:**
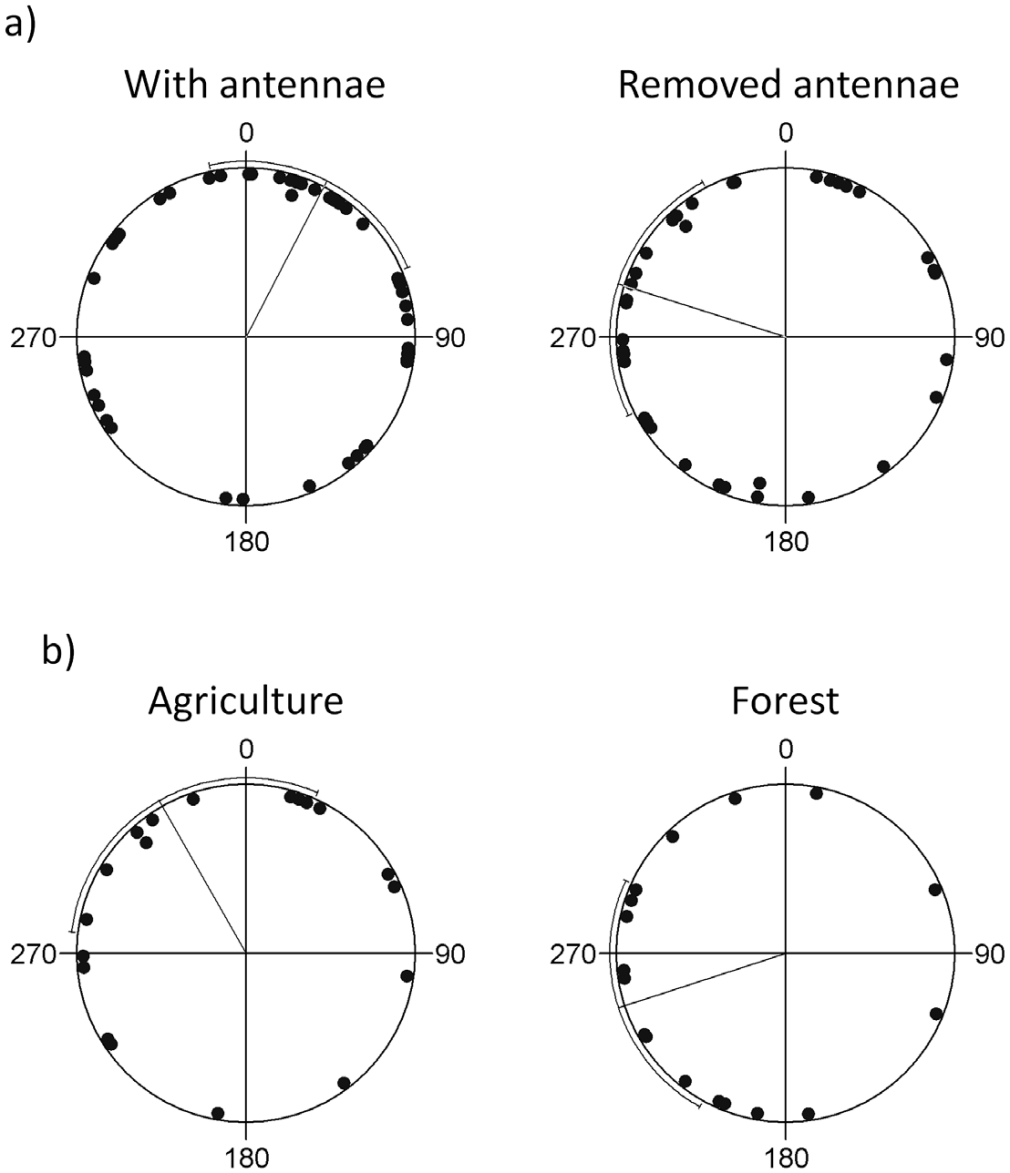
Flight directions at take off in a) control butterflies (with antennae) compared to those deprived of antennae, and b) butterflies deprived of antennae originating from agricultural and forest landscapes. The direction to the target habitat is set to 0°. The circumferential error bars represent 95% confidence intervals. Butterflies with antennae and those from fragmented agricultural landscapes were more likely to orient towards the target habitat than those of a forest origin and deprived of antennae.

## Materials and Methods

### Populations and Breeding

Speckled wood butterfly *Pararge aegeria* breeding populations from two forested landscapes and two agricultural landscapes were established in the laboratory. All sampled populations were located in Belgium (Forested landscapes: Meerdaalwoud: 50°80′ N, 4°70′ E and Arville: 50°05′ N, 5°30′ E – Agricultural landscapes: Lillois: 50°64′ N, 4°36′ E and Morkhoven: 51°11′ N, 4°82′ E). Standardized conditions for photoperiod, temperature and host grass (i.e. *Poa trivialis*) resulted into direct larval development. Pupae were removed from the grass and kept individually in plastic containers until emergence. One day after eclosion, butterflies were weighted, individually marked on their wings and placed in small cages for mating. They had access to 5% honey solution. After mating, adults were stored in a cold room at 10°C until they were used in the field experiment. *P. aegeria* is not a legally protected species according to Belgian and international conservation legislation. Under Belgian legislation, there are no ethical policies that apply to experiments on wild insects like *P. aegeria*.

**Table 1:** Average flight directions of released intact (with antennae) Speckled wood butterflies *Pararge aegeria* L. and butterflies from which the antennae had been removed immediately after take off.

	With antennae			Removed antennae				
	Direction(°)	V-test		Direction(°)	V-test		Watson’s U^2^-test	
		V	P		V	P	U^2^	P
**All**	27	0.256	0.006	288	0.089	0.23	0.198	<0.05
**25 m**	42	0.319	0.021	276	0.041	0.40	0.267	<0.01
**50 m**	5	0.209	0.063	310	0.137	0.208	0.044	>0.5
	**Removed antennae**							
	**Forest**			**Agricultural**				
	252	–0.134	0.78	330	0.288	0.038	0.123	>0.10

V-tests to test for the directedness and Watson’s U^2^-tests for pair-wise comparisons between groups. A P-value lower than 0.05 in the V-test indicates that the mean flight direction *is not* significantly different from 0°, i.e. the direction towards the target habitat.

### Release Experiment

To mimic the situation faced by a butterfly searching for habitat in an unfamiliar, homogeneous open landscape matrix, we conducted a field experiment were we released naïve adult male and female butterflies from the laboratory stock in an open field at fixed distances from a habitat patch and recorded the flight path of each individual [19]–[20].

Butterflies (N = 201, 91 from forested landscapes, 110 from agricultural landscapes; 95 males and 106 females) were released on one of two open fields at 50 (N = 94) or 100 m (N = 107) from a target habitat in an agricultural landscape near Gembloux, Belgium (50° 34′ N, 4° 41′ E). Release experiments were done in September 2009 (9 days with suitable weather conditions that allow butterfly flight activity (i.e. sunshine, ambient temperature >20°C; wind speed <5 m/s)). At both sites, the target habitat consisted of a tall, dense hedgerow (height: 6 m and 15 m, resp.). Earlier experiments have indicated a similar preference for woody landscape elements for butterflies of forested landscape and of agricultural landscape [18]–[19]. Butterflies were transported to the release site in a cool box. Each butterfly was transported in an individual plastic cup covered with a fine-meshed net. Butterflies were released one by one from the plastic cup by removing the net and allowing the butterfly to take off spontaneously. Each individual was released only once; release order and distance was randomized relative to the groups of interest (landscape of origin and sex). Butterflies not flying from the release point within five minutes were classified as “not flying”, and were removed from the release point and excluded from any further experiments.

Individual flight paths were tracked at a distance of *c*. 2 m by one person with a hand-held GPS (Garmin eTrex Legend HCX). Positions were recorded every second. An observation session lasted until the butterfly either: 1) reached the target habitat; 2) did not reach habitat but flew more than 100 m away from the point of release and continued to fly into the open, agricultural landscape away from the forested habitat; 3) interrupted its flight and rested more than 2 min; or 4) was lost out of sight.

### Release without Olfactory Cues

To test whether olfactory cues are of any significance for oriented flight to a target habitat, we carefully removed the antennae [21] of 40 females of either forest or agricultural landscape origin (N = 20 for each type; these butterflies were not included in the first release experiment). They were released at 25 m or 50 m distance from the target habitat. Their responses were compared against untreated females released at both distances (N = 22 and N = 26, resp.).

As a further control treatment, we removed a single antenna from 10 female butterflies. The release and observation procedure followed the description for the main experiment above.

### Analyses

The probability of an individual flying from the release point or not, relative to landscape of origin, was tested by a Generalized Linear Mixed Model with binomial error distribution (SAS Proc Glimmix) [22]. Landscape of origin, age (days since emergence), body mass, sex, release site and release distance, and all two-way interactions were included as predictor variables. Family, defined as the offspring of a single female, nested within population was included as a random factor. 191 individuals were included in this model. The final model was obtained by backward selection. We used a similar model to test for variables that influenced the probability of reaching the target habitat patch. This model included only the butterfly individuals that left the release point within five minutes (N = 159).

Although several butterflies flew towards the habitat already from take-off, other ones started with small loops in different directions or with a zigzag pattern, and initiated only after some time a directed flight towards the target habitat. Based on the total flight paths, we classified each individual as either a) directed towards the habitat from take off, b) never reaching the habitat, or c) not flying towards the habitat from take off, but eventually reaching the habitat. For the latter category, we measured at what distance from the habitat the straight flight was initiated. We fitted a GLMM (SAS Proc Mixed) with this distance as the response variable and predictor variables, random factors and selection procedure as above. 49 individuals were included in this model.

We analyzed directedness towards the target habitat using circular statistics using Oriana 3.11 [23]. We used the initial direction rather than the mean or final direction in our analyses in order to avoid problems with null models [24]. We used V-tests [25] to test whether flight angles for a specified subset of butterflies were on average directed towards the target habitat (specified as 0°). Differences of the distribution of flight directions between groups were tested by Watson’s U^2^-test [25]. The analyses of willingness to fly, probability to reach the target habitat and flight orientation described above were repeated for treated (antennae removed) vs untreated butterflies. The independent variables used were release distance (25 or 50 m), butterfly age, weight, landscape of origin and treatment, and their two-way interactions. As relatively few butterflies deprived of antennae reached the target habitat, we could not compare treated and control butterflies with respect to the linearity of flight paths.

## Results

### Release Experiment

Among the 201 released butterflies, 42 did not fly from the release point within 5 minutes. The probability of an individual leaving the release point and flying through the hostile matrix decreased with age of the butterfly (*β = −0.15* day^−1^, *F*_1,147_ = 17.0, *P*<0.001) and was higher for males (percentage of individuals flying (±SE) = 84±3.8%) than for females (75±4.2%; *F*_1,147_ = 7.19, *P* = 0.008). There was, however, no significant difference between butterflies of different landscape origin (agricultural: 81±3.8%; forest 77±4.4%; *F*_1,147_ = 1.44, *P* = 0.23) and no effect of release distance (*F*_1,146_ = 0.17, *P* = 0.68) or site (*F*_1,146_ = 1.22, *P* = 0.27). None of the two-way interactions was statistically significant.

Of the 159 butterflies that left the release point, 112 (i.e. 70%) reached the target habitat. The probability of reaching the target habitat was higher for butterflies released at 50 m than at 100 m from the target habitat (*F*_1,98_ = 5.58, *P* = 0.02), decreased with butterfly age (*F*_1,98_ = 7.50, *P* = 0.007) but did not differ between butterflies of forest and agricultural landscape origin (*F*_1,98_ = 0.88, *P* = 0.35). There was no effect of sex (*F*_1,98_ = 2.22, *P* = 0.14) or release site (*F*_1,98_ = 0.25 *P* = 0.62).

The average flight path among all butterflies was directed towards the target already at take off (V = 0.322, P>0.001). There was, however, a significant difference in the distribution of flight angles at take off between butterflies that eventually reached the habitat and those that did not (*U^2^* = 0.312, *P*<0.005). Butterflies that reached the target habitat were heading in the direction of the habitat already at take-off (*V* = 0.426, *P*<0.001), whereas butterflies not reaching target habitat were flying in randomly distributed directions (*V* = 0.029, *P* = 0.40).

Among the 112 butterflies that reached the target habitat, 50 (45%) were flying in a straight line towards the habitat already from take off, whereas 55% were first flying in a zigzag pattern or making petal-like loops around the release point [19] before initiating a straight flight towards the habitat. In the latter category, individuals originating from agricultural populations initiated a linear flight towards the habitat at a greater distance from the target habitat compared to individuals from forest populations (*F*_1,22_ = 4.85, *P* = 0.039, Fig. 1, irrespective of release distance (interaction landscape × distance: *F*_1,20_ = 1.95, *P* = 0.18).

### Release without Olfactory Cues

The probability of reaching the target habitat was significantly lower for butterflies which had their antennae removed (41±8.1%) than for control butterflies (81±4.3%; *F*_1,47_ = 9.94, *P* = 0.003), and tended to be lower for butterflies of forest origin (55±6.3%) than for those originating from agricultural populations (73±8.0%; *F*_1,47_ = 3.39, *P* = 0.072), but there was no interactive effect of treatment and origin.

Only butterflies with intact antennae, and not those with removed antennae were on average directed towards the habitat at take off (Table 1; Fig. 2a). Among butterflies deprived of antennae, the ones with an agricultural population origin, but not those originating from forest populations, were on average oriented towards the habitat (Fig. 2b).

Butterflies with a single antenna removed did not differ in their willingness to fly (*F*_1,20_ = 0.02, *P* = 0.88) or orientation (U^2^ = 0.157, P>0.05) from control butterflies. Butterflies with a single antenna removed (V = 0.558, P = 0.008) but not those with both antennae removed (V = 0.124, P = 0.15) were significantly directed towards the target habitat.

## Discussion

The ability of species to adapt their search strategies following environmental changes such as habitat fragmentation is likely to significantly reduce their dispersal cost and thereby increase their chances of survival in intensively modified landscapes. Our observation that butterflies originating from agricultural and forest landscapes on average initiated a linear flight at different distances towards the target habitat, in combination with the observation that butterflies from agricultural landscapes had a higher probability to reach the target habitat than butterflies from forest landscapes if their antennae were removed indicate intrinsic differences in habitat finding abilities among individuals that originate from different types of landscape. A longer perceptual range in butterflies from agricultural populations is in line with adaptive predictions of improved habitat finding ability in fragmented landscapes, which leads to a reduced dispersal cost [4], [26]. Hence, we can confirm our earlier preliminary experiments with only wild-caught *P. aegeria* males from one forested and one agricultural landscape population [19], but now we can exclude that the observed differences only resulted from different environmental experience. Our common garden approach suggests a heritable basis for the differences in habitat finding ability. Further work needs to reveal whether genetic differences or adaptive maternal effects among populations explain the differences between butterflies of the two landscape types [27]. *P. aegeria* butterflies from continuous forested landscapes and fragmented agricultural landscapes have recently been shown to differ phenotypically and genetically in several other behavioural, morphological and life-history traits (e.g. 27–31]. It is thought that high levels of phenotypic plasticity in life history traits and functional morphology in this mutivoltine species represent a significant survival advantage in highly dynamic anthropogenic environments [32].

Our next step was to explore the proximate mechanism of the difference in habitat finding ability by manipulating access to olfactory information. Our finding that the majority of (untreated) butterflies were flying towards the target habitat already at take off from the ground, before they had any chance to get a visual image of the surroundings suggests that olfactory information plays an important role in locating suitable habitats.

The probability of reaching the target habitat was significantly lower for butterflies which had their antennae removed. Among butterflies deprived of antennae, the ones with an agricultural population origin, but not those originating from forest populations, were flying towards the habitat already at take-off. These results suggest that butterflies use a combination of visual and olfactory information to locate suitable habitat patches, but that butterflies originating from populations in agricultural landscapes were better able to compensate for the loss of one type of information, i.e. olfactory cues. An alternative explanation is that it is an adaptive strategy to rely more on visual cues in agricultural than in forested landscapes. In forested habitats, the visual range is typically much shorter than in open landscapes, so olfactory information might be more reliable to detect distant objects. This needs further experimental analysis.

The distance at which animals can detect suitable habitat patches and other landmarks has implications for the functional connectivity of landscapes [33]–[34]. Including perceptual range in dispersal and metapopulation models could potentially have large effects on predicted immigration, emigration and population persistence probabilities [35]. A wide perceptual range could reduce the cost of dispersal and diminish the impact of the spatial arrangement of habitat patches on population dynamics [33]–[35]. The mode of perception can have consequences for predicting the functional connectivity of a landscape. In animals mainly using olfactory information when locating suitable habitats, the perceptual range will be asymmetric and depend on wind conditions, i.e. the perceptual range is wider upwind than downwind [33]. As a result, the functional connectivity will be asymmetric. This is, however, rarely accounted for in metapopulation models.

Our findings suggest that the functional connectivity of a landscape, and hence the dispersal cost, differs between individuals of the same species depending on their landscape of origin. Such between-population differences need to be considered when predicting how species will shift their distributions in response to climate change. Previous studies have suggested that climate-induced distribution shifts might be slower in more fragmented landscapes [36]. Our results suggest that adaptation to reduce dispersal costs can at least to some extent compensate for such negative effects of habitat fragmentation.
